# Supplementary material for: Hybrid low-voltage physical unclonable function based on inkjet-printed metal-oxide transistors
Source: Nat Commun. 2020 Nov 2;11:5543. doi: 10.1038/s41467-020-19324-5 (PMC7608659; doi:10.1038/s41467-020-19324-5)
Supplement: Supplementary file 1 — Supplementary Information [file 41467_2020_19324_MOESM1_ESM.pdf]

Supplementary Information

Hybrid low-voltage physical unclonable function based on inkjet-printed metal-oxide transistors

Scholz et al.

Supplementary Note 1

Functionality of the design

The hardware design is split into three functional units, each located on a separate PCB. Supplementary Fig. 1 shows the hybrid PUF platform and measurement system, consisting of the (a) development board, (b) control logic, and the (c) PUF core adapter PCB hosting the printed core circuit. The flexible design allows the functional units to be interchanged, enabling large-scale characterization of printed PUF cores at reduced costs and production times.

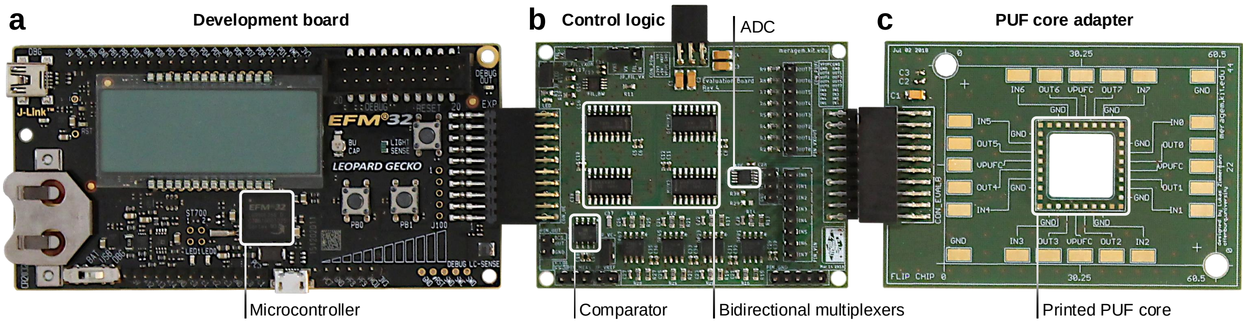

Supplementary Figure 1: Hybrid PUF platform and measurement system. **a** The EFM32 development board, **b** control logic, **c** PUF core adapter PCB module with mounted printed PUF core glass substrate (from left to right). Due to the transparency of the ITO strips, they are not visible in this image.

The printed PUF core consists of eight EGT resistor logic inverters. The control logic circuitry is dimensioned correspondingly to enable arbitrary addressing of the PUF core inverters' inputs and outputs. The design provides non-linear scaling of the number of possible PUF core inverter address permutations, similar to ring oscillator PUFs. Supplementary Fig. 8 in the Appendix shows photos of the PUF core adapter PCB, the printed PUF core, and microscope images of the printed EGTs. The microscope images visualize the variations caused by the printing process.

PUF Test cases

We apply the test cases P_1 to P_{11} shown in Supplementary Table 1 for evaluating the security metrics on our fabricated hybrid PUFs. In general, each distinct test case covers a unique combination of operating conditions, including different ambient temperatures (T_{amb}), relative humidities (RH), and the PUF core supply voltages ($\mathrm{VDD}_{\mathrm{core}}$). Based on the requirements for computing the corresponding PUf security metric, different test cases are required. For printed PUF uniqueness evaluation, test case P_1 is used. To evaluate the bit errors of the printed PUF, test cases P_1, P_6 and P_9 are used. The reliability metric is evaluated with test cases $P_1 - P_{11}$.

Comparison of PUFs

We qualitatively compare this work with other state of the art experimentally evaluated PE-based PUFs. To the best of our knowledge no fully verified PE-based PUF including the evaluation of all security metrics has been reported in literature, yet. However, evaluating the PUF security metrics is important to enable a qualitative comparison between different PUF implementations. Nonetheless, currently existing comparable results are listed in Supplementary Table 2. We compare the used printing technology, the type of response generation (electrical or optical), the PUF type (weak or strong PUF), the presented response bit width, as well as experimentally verified PUF security metrics. This includes the uniqueness, reliability, bit aliasing, bit errors, false-acceptance-rate (FAR), and false-rejection-rate (FRR).

Supplementary Table 1: Test cases for hybrid PUF experiments (test cases marked with × denote the configured test setup P_w).

**Test case**	**Ambient temperature**			**Rel. humidity**			**VDD_{core}**		
P_w	20 °C	40 °C	60 °C	45 %	50 %	55 %	0.9 V	1.0 V	1.1 V
P_1	×				×			×	
P_2	×			×				×	
P_3	×					×		×	
P_4	×				×		×		
P_5	×				×				×
P_6		×			×			×	
P_7		×			×		×		
P_8		×			×				×
P_9			×		×			×	
P_{10}			×		×		×		
P_{11}			×		×				×

Supplementary Table 2: Qualitative comparison of PE-based PUF implementations. The comparison includes experimentally verified PUFs. The absence of a compared PUF parameter is denoted by a (-).

**PUF**	**Printing technology**	**Response generation**	**PUF type**	**Response bit width**	**PUF metrics (experimental)**	**Suppl. Ref.**	**Year**
Memory-PUF	Inkjet	Electrical	Weak	1	-	[1]	2018
Resistive CNT-PUF	Inkjet	Electrical	Weak	-	-	[2]	2019
SRAM-PUF	Screen	Electrical	Weak	4	-	[3]	2012
Quantum Dot-PUF	Inkjet	Optical	Strong	-	-	[4]	2019
**This work (Hybrid PUF)**	**Inkjet**	**Electrical**	**Weak**	**28**	**Uniqueness Reliability Bit error Bit aliasing FRR and FAR**		**2020**

Challenge-response behavior

A full readout PUF challenge contains 28 unique pairs of inverter addresses, which are given by the permutation. The single address pairs are called sub-challenges, whereas the resulting response bits are called sub-responses.

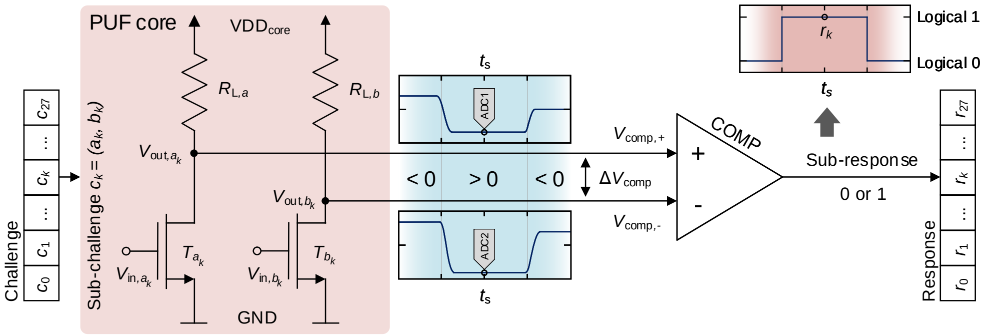

Supplementary Figure 2: Hybrid PUF platform signal path for response generation. The PUF challenge divides into 28 sub-challenges that are successively applied to the PUF. The sub-challenge c_k, shown as an example, includes the inverter address tuple (a_k, b_k). The comparator (COMP) generates the digital sub-response bit r_k, based on its positive (+) and negative (−) input terminals.

The complete PUF response as an identifier is generated by successively applying the sub-challenges to the PUF. Supplementary Fig. 2 shows how challenges are applied to the PUF and responses are generated. The k-th sub-challenge c_k includes the inverter address tuple (a_k, b_k). The inverter input biasing voltages $V_{\mathrm{in},a} = V_{\mathrm{in},b}$ are equal and remain stable during response generation. The comparator (COMP) generates digital sub-response bits based on its positive (+) and negative (−) inputs $V_{\mathrm{comp},+}$ and $V_{\mathrm{comp},-}$, respectively. In addition to the digital response bit, it is also possible to measure the corresponding analog voltage levels at the comparator input terminals with a 12-bit ADC. To reduce ADC quantization errors, averaging over eight measurements is done. The minimum resolvable signal by the ADC is 500 µV.

Response extraction

To evaluate the fabricated hybrid PUFs, we apply the challenge C and measure the PUF response R', as shown in Supplementary Fig. 3a. Additionally, we utilize a BCH error correcting code (ECC) to compute the syndrome for each response. Supplementary Fig. 3b shows the extraction phase, which is used to generate PUF responses for evaluation purposes. In general, the response R' is expected to be instable, for what reason we apply a BCH error correction, leading to the stable response R.

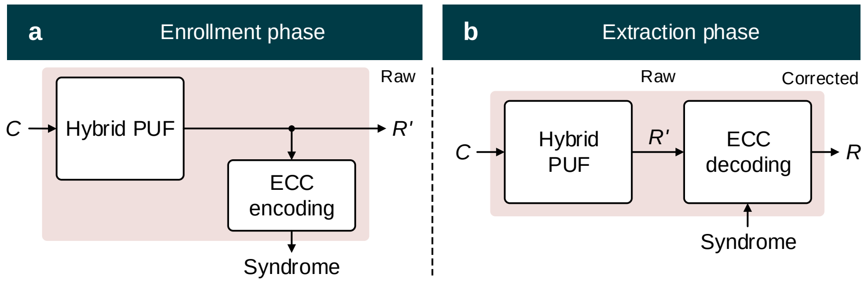

Supplementary Figure 3: Hybrid PUF response generation. **a** Enrollment phase: PUF response extraction R' (raw) and syndrome computation by the error correcting code (ECC). **b** Extraction phase: PUF response extraction R' and error correction using the syndrome for a stable response R (corrected).

The following protocol is used to generate the data basis that is needed to investigate the hybrid PUFs:

1. Enrollment phase: Under nominal conditions, a fixed challenge is applied to each hybrid PUF and the resulting 28-bit PUF response is measured. The challenge-response-pairs (CRPs) are stored in a database. The reference response of each hybrid PUF is determined by the majority voting of 20 repeated measurements.

2. Extraction phase: The fixed challenges are applied again to the hybrid PUFs, while being exposed to different operating conditions. The resulting CRPs are stored in a database.

3. Error correction: To mitigate bit flips resulting from changing operating conditions, an error correction based on BCH codes is applied.

4. Evaluation phase: For the collected CRPs, the intra- and inter-hamming distances (HDs) are computed to evaluate the associated distributions with respect to identification error probabilities.

Entropy of the PUF responses

To determine the entropy of random numbers the min-entropy estimation is widely used, as recommended in the NIST specification 800-90[1]. The min-entropy metric is based on the distribution of 0s and 1s. Thereby, the probability of having a 0 is denoted by p_0, whereas the probability of a 1 is p_1. In general, the min-entropy $H_{\min}$ is calculated according to Equation(1):

[1]Barker, E., Feldman, L., & Witte, G. (2015). *Recommendation for random number generation using deterministic random bit generators (No. ITL Bulletin August 2015).* National Institute of Standards and Technology.

$$H_{\mathrm{min}} = -\log_2(p_{\mathrm{max}}) \tag{1}$$

where $p_{\mathrm{max}} = \max(p_0, p_1)$. In the context of PUFs the min-entropy metric considers the bias of the PUF responses. As shown in Fig. 4a in the manuscript, the average bit aliasing, which corresponds to the bias, is $\mu_m = 44.5\,\%$ for the fabricated and evaluated hybrid PUFs. This leads to a min-entropy of $H_{\mathrm{min}} = -\log_2(0.555)) = 0.849$, which denotes that the actual information content of a 28-bit response is limited to ≈ 23.772 bits. However, for our simulation results we determined a mean bit aliasing value of $\mu_{\mathrm{m}} = 49.8\,\%$, which leads to a min-entropy value of $H_{\mathrm{min}} = -\log_2(0.502) = 0.994$. This value indicates the theoretical performance that can be expected for greater sample sizes in our approach.

Hybrid PUF identification

To investigate the identification capabilities of the hybrid PUF, we perform evaluations based on the intra-HD and inter-HD distributions. The intra-HD is a measure of the reproducibility of the PUF responses for a fixed challenge and under the impact of changing operating conditions. The inter-HD indicates the uniqueness of the responses generated by different PUFs. Supplementary Fig. 4a shows the intra- and inter-HD distributions for our measured PUF responses under humidity and voltage variations. The solid black line shows the intra-HD Gaussian distribution, whereas the dash-dot red line shows the inter-HD Gaussian distribution, respectively. The enclosed area below both lines divides into the two regions left and right from the intersection value. The left region is denoted as the false-acceptance rate (FAR), whereas the right one is the false-rejection rate (FRR). To ensure a proper identification, both the FAR and FRR should be minimized and the PUF responses should contain enough entropy with respect to the sample size. As there is an overlap between both, the intra- and the inter-HD variation distributions, some PUFs cannot be distinguished without additional post-processing.

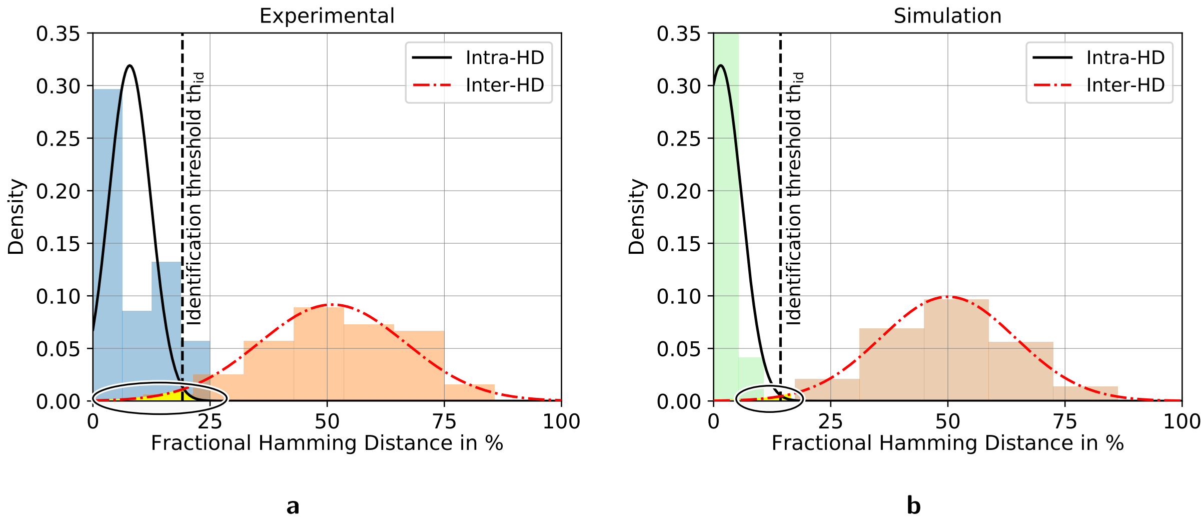

Supplementary Figure 4: Intra- and inter-hamming distance (HD) distributions. **a** Gaussian distributions based on our measured data. **b** Gaussian distributions based on simulated data.

The x-value of the intersection is the ideal threshold value to distinguish between PUF responses when applying a binning technique. If the HD between the database entry and the measured response is less than this threshold, the identification is successful. If there is no overlap between both distributions, the identification can be assumed errorless, if the threshold is placed somewhere in between.

Supplementary Fig. 4b shows the corresponding intra- and inter-HD Gaussian distributions for the simulated data of 150 printed PUF cores. The simulation data used has been generated in our prior work and refers to the worst case considerations where a noise level of 10 mV is applied. The standard deviation of

the inter-HD can be expected to decrease for larger sample sizes. The overlap in the plot between both distributions is small, which implies a low identification error of the hybrid PUFs. A three bit error correction reduces the intra-HD to zero and also eliminates the overlapping area. To further assess the identification capabilities, based on the raw PUF responses (without additional error correction), we compute the FAR and FRR values based on experimental and simulation data. The FAR and FRR values are calculated according to Equation (2) and Equation (3), respectively:

$$\mathrm{FAR} = \int_0^{\mathrm{th_{id}}} P_{\mathrm{inter}}(x)\ dx \tag{2}$$

$$\mathrm{FRR} = \int_{\mathrm{th_{id}}}^{L} P_{\mathrm{intra}}(x)\ dx \tag{3}$$

where $P_{\mathrm{inter}(\cdot)}$ and $\mathrm{P_{intra}}(\cdot)$ are the probability density functions of the inter-HD and intra-HD distributions. Since the FAR and FRR values are typically very small numbers, it is common practice to use the $\log_{10}(\cdot)$ representation. Typical FAR and FRR values used in identification systems reach from -3 up to -12 (after post-processing). Basically, the FAR and FRR values depend on the selected identification threshold value. Two often used approaches to set the identification threshold is (1) to use the intersection point between both distributions and (2) use the so-called equal-error-rate (EER) where FAR=FRR. We use the experimental response data and calculate the FAR and FRR values for the former identification threshold. The resulting values are FAR=-2.23 and FRR=-1.71. Furthermore, we compute the values for the EER, which result in FAR=FRR=-1.83. To the best our knowledge, this is the first assessment of the identification capabilities of a PE-based PUF. Even in the more matured research field of silicon-based PUFs, such detailed statistical evaluations are rare. At this point we want to note that our evaluations are based on raw PUF responses without additional post-processing, such as error-correction. For our simulation data, the resulting values are FAR=-2.21 and FRR=-2.68. For the EER the values are FAR=FRR=-2.32. The results show that the experimental results are in good agreement with our simulations. However, additional post-processing could further improve the identification capabilities of the hybrid PUF.

Increasing the challenge space

The simulated inverter voltage transfer curves, as shown in Supplementary Fig. 5, provide a different slope per device at various input voltage levels V_{in} due to intrinsic variation. Therefore for EGTs, some voltage transfer curves change their corresponding voltage difference at various input voltage levels.

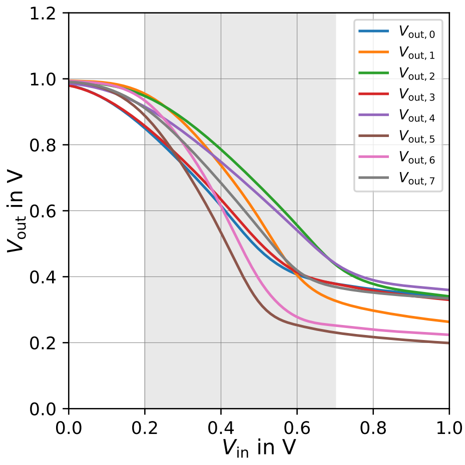

Supplementary Figure 5: Simulated voltage transfer curve with eight inverters.

This property can be exploited and allows for an expansion of the sub-challenge along V_{in} with the tuple (a_k, b_k), according to $(V_{\text{in}}, a_k, b_k)$. Now we can extract slightly different responses for the same addressed inverters over various input voltage levels with low overhead, as shown in Supplementary Table 3. However, as discussed before, when biasing at high (1 V) and very low input voltages (0 V), the voltage differences are usually smaller than at the best operating point $V_{\text{in}} = 0.4\,\text{V}$.

Supplementary Table 3: Hybrid PUF responses with same challenge over different V_{in}.

V_{in}	Hybrid PUF response
0 V	0010000111111111010000000011
0.1 V	0000000111111111010000000001
0.2 V	0000000111111111110000101001
0.3 V	0000100011111111110100111000
0.4 V	0000110010111111110110111000
0.5 V	0000110010111111110110111000
0.6 V	1000110000110111110110111000
0.7 V	1010111000110101110111111000
0.8 V	1010111000110101110111111000
0.9 V	1010111000110101110111111000
1.0 V	1010111000110101110110111000

Supplementary Note 2

Printed PUF core analog signal level characteristics

In addition to Fig. 3d in our regular paper, we also show the plots for the voltage differences on the comparator inputs at the ambient temperatures $T_{\mathrm{amb}} = 40\,°\mathrm{C}$ and $T_{\mathrm{amb}} = 60\,°\mathrm{C}$ in Supplementary Fig. 6a and 6b, respectively. Supplementary Fig. 6c also shows the comparator input voltage differences over the ambient temperatures $T_{\mathrm{amb}} = \{20\,°\mathrm{C}, 40\,°\mathrm{C}, 60\,°\mathrm{C}\}$.

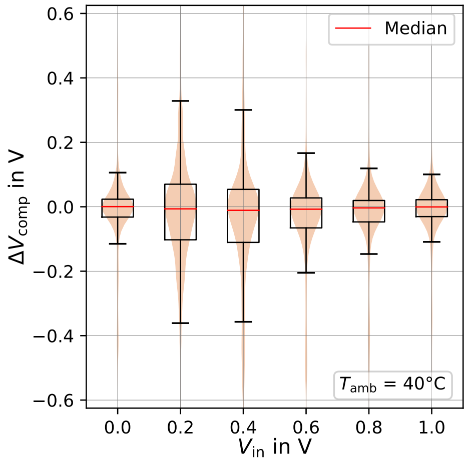

a

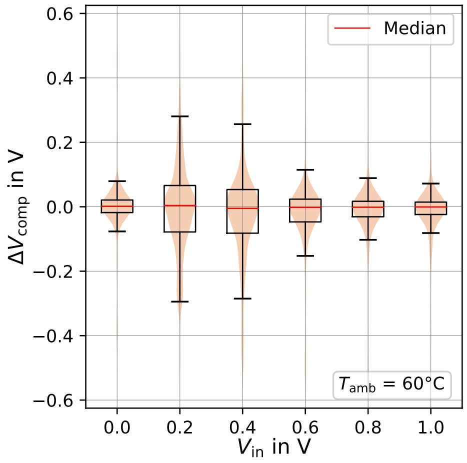

b

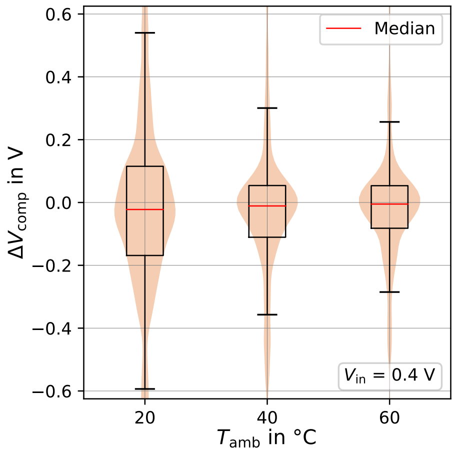

c

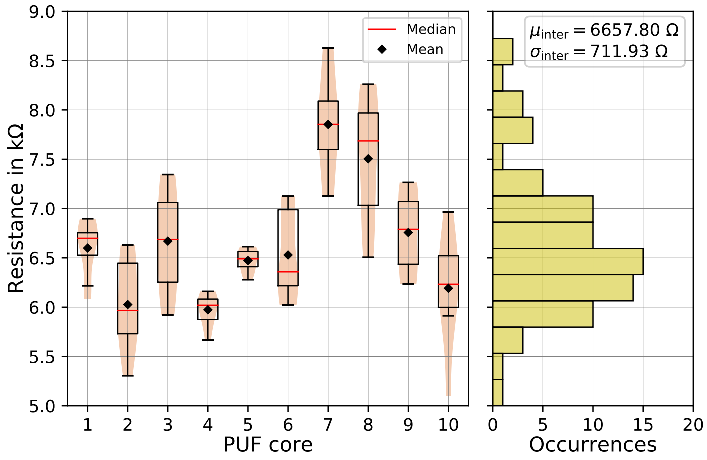

d

Supplementary Figure 6: **a** Voltage difference levels ΔV_{comp} at the comparator input terminals including all PUF core inverter pair combinations at the ambient temperature 40 °C. **b** Voltage difference levels ΔV_{comp} at the comparator input terminals including all PUF core inverter pair combinations at the ambient temperature 60 °C. **c** Voltage difference levels ΔV_{comp} at the comparator input terminals at 0.4 V input voltage over $T_{\mathrm{amb}} = \{20\,°\mathrm{C}, 40\,°\mathrm{C}, 60\,°\mathrm{C}\}$ **d** Resistance variation over 10 PUF core instances, with inter-substrate variation over R_L on the left side, and intra-substrate variation R_L on the right side.

Load resistors

To gain insight into the ITO structuring process, which is performed using laser ablation, we measured the corresponding resistance values of the meander-structured ITO strips. Supplementary Fig. 6d shows the resistance variations of the fabricated PUF core resistors. A Keithley 2000 digital multimeter is used for measurement acquisition. The measured mean resistivity is $6.632\,\mathrm{k\Omega}$ with a standard deviation of $\sigma = 0.737\,\mathrm{k\Omega}$. The violin boxplot (left) in Supplementary Fig. 6d shows the intra-substrate resistance variations of ten fabricated PUF cores. In comparison, the histogram (right) shows the inter-substrate resistance variations. The intra-substrate variations are smaller but the mean values differ between the cores, which leads to a broad inter-substrate resistance distribution. The variation is caused by the laser ablation process, which requires manual adjustment of the base plate for focusing. Furthermore, the resistance measurements are performed with a multimeter, which also introduces variations due to manual needle placing on the resistive elements.

Supplementary Note 3

Simulation workflow

To validate our measurements of the fabricated hybrid PUFs, we show the simulation results of the uniqueness and bit aliasing metrics gathered from our prior work in Fig. 4a and 4b of the paper manuscript. The simulations were performed on an EKV-based semi-physical model for inkjet-printed EGTs. In addition, a software tool was implemented to emulate the silicon-based addressing and bit generation logic.

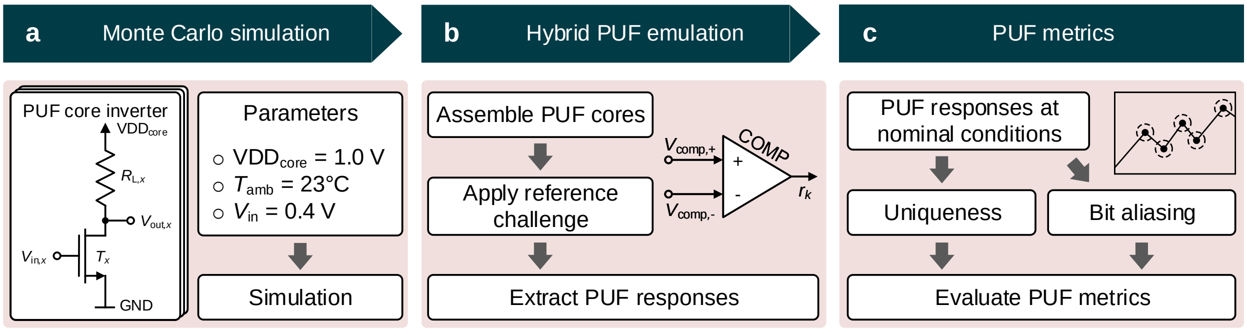

Supplementary Figure 7: Monte Carlo simulation and hybrid PUF evaluation workflow.

Supplementary Fig. 7 shows the workflow used for the Monte Carlo (MC) simulations and hybrid PUF emulation. In the first step (a), we performed MC simulations on PUF core inverters at nominal operating conditions ($\mathrm{VDD_{core}} = 1.0\,\mathrm{V}$, $T_{\mathrm{amb}} = 23\,°\mathrm{C}$, $V_{\mathrm{in}} = 0.4\,\mathrm{V}$) and created 1,200 samples. In (b), we use our specially implemented Python-based software emulation tool to virtually assemble PUF cores from the MC samples. In the next step, we apply the same challenge to the emulated PUFs as we did for the fabricated hybrid PUFs and store the corresponding PUF responses. Finally (c), we evaluate the PUF responses regarding the uniqueness and bit aliasing metrics. The results are shown in Fig. 4a and 4b in the paper, respectively.

Supplementary References

[1] Erozan, A. T. et al. Inkjet-printed EGFET-based physical unclonable function - design, evaluation, and fabrication. *IEEE Trans. Integr. Syst.* **26**, 2935–2946 (2018).

[2] Moon, D.-I. et al. Physically unclonable function by an all-printed carbon nanotube network. *ACS Appl. Electron. Mater.* **1**, 1162–1168 (2019).

[3] Guerin, M. et al. Design of organic complementary circuits for RFID tags application. in *2012 IEEE Custom Integrated Circuits Conference*, 1-4 (IEEE, 2012).

[4] Liu, Y. et al. Inkjet-printed unclonable quantum dot fluorescent anti-counterfeiting labels with artificial intelligence authentication. *Nat. Commun.* **10**, 2409 (2019).

Appendix

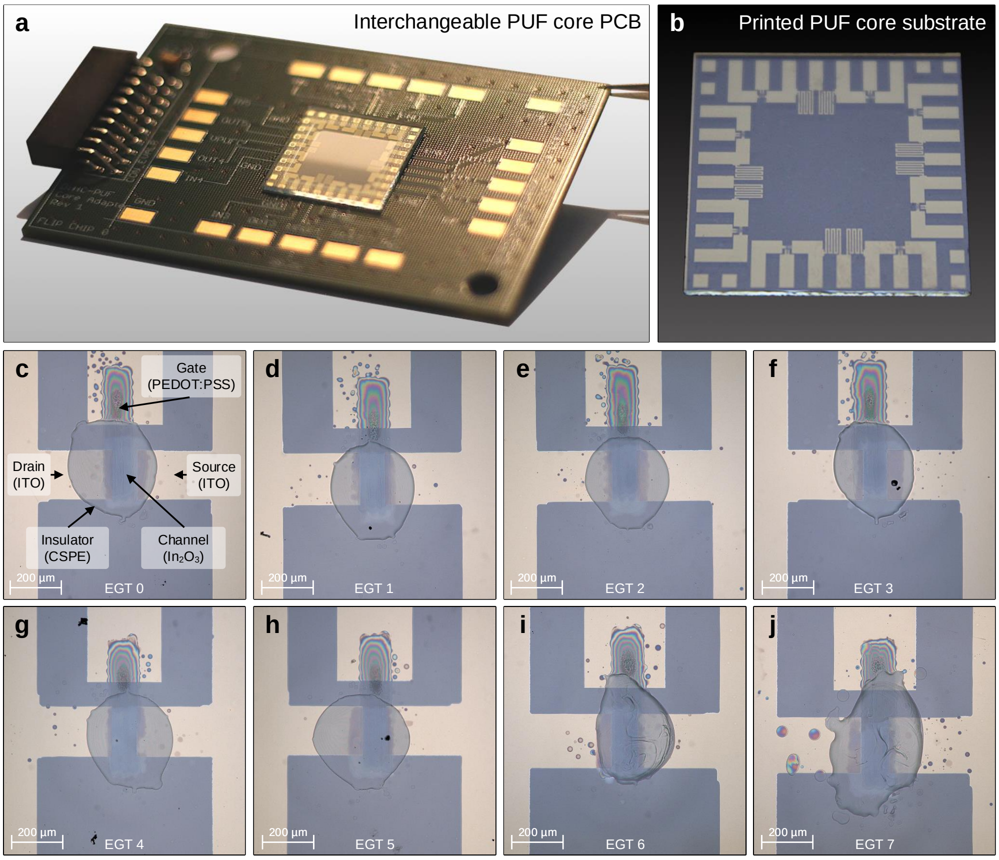

Supplementary Figure 8: Interchangeable PUF core adapter PCB. **a** The printed PUF core glass substrate mounted onto the adapater PCB. **b** Detailed view of the printed PUF core glass substrate. **c-j** Microscope images of the eight EGTs on a PUF core.
